# Bladder cancer-derived interleukin-1 converts the vascular endothelium into a pro-inflammatory and pro-coagulatory surface

**DOI:** 10.1186/s12885-020-07548-z

**Published:** 2020-12-02

**Authors:** A. John, C. Günes, C. Bolenz, S. Vidal-y-Sy, A. T. Bauer, S. W. Schneider, C. Gorzelanny

**Affiliations:** 1 Department of Urology, University of Ulm, Ulm, Germany; 2 Department of Dermatology, University Medical Center Hamburg-Eppendorf, Martinistraße 52, 20246 Hamburg, Germany

**Keywords:** Tumour microenvironment, von Willebrand factor, Coagulation, Inflammation, Endothelial cells

## Abstract

**Background:**

Bladder cancer cells orchestrate tumour progression by pro-inflammatory cytokines. Cytokines modulate the local tumour microenvironment and increase the susceptibility of tumour distant tissues for metastasis. Here, we investigated the impact of human bladder cancer cell derived factors on the ability to modulate and activate human vascular endothelial cells.

**Methods:**

The pro-inflammatory and pro-coagulatory potential of four different bladder cancer cell lines was accessed by qRT-PCR arrays and ELISA. Modulation and activation of endothelial cells was studied in microfluidic devices. Clinical relevance of our findings was confirmed by immune histology in tissue samples of bladder cancer patients and public transcriptome data.

**Results:**

The unbalanced ratio between interleukin (IL)-1 and IL-1 receptor antagonist (IL-1ra) in the secretome of bladder cancer cells converted the quiescent vascular endothelium into a pro-adhesive, pro-inflammatory, and pro-coagulatory surface. Microfluidic experiments showed that tumour cell induced endothelial cell activation promoted leukocyte recruitment and platelet adhesion. Human bladder cancer tissue analysis confirmed that loss of IL-1ra and elevated IL-1 expression was associated with enhanced cancer progression.

**Conclusions:**

Our data indicate that IL-1 and IL-1ra were dysregulated in bladder cancer and could facilitate tumour dissemination through endothelial cell activation. Targeting the IL-1/IL-1ra axis might attenuate tumour-mediated inflammation and metastasis formation.

**Supplementary Information:**

The online version contains supplementary material available at 10.1186/s12885-020-07548-z.

## Background

Advanced urothelial bladder cancer (UBC) is characterized by poor prognosis and a median survival of only 14 months after first line chemotherapy with gemcitabine and cisplatin [1]. High metastatic potential and limited treatment alternatives for patients not eligible for or refractory to platinum-based combination chemotherapy present major therapeutic challenges. Although immunomodulatory therapies using checkpoint inhibition, present promising options in metastatic disease, their administration can induce severe autoimmunity related side effects and response rates are in the range of only 20–30% [2].

Tumour progression is linked to local and systemic pro-inflammatory and pro-thrombotic intravascular conditions [3]. Consequently, risk of thromboembolism is high in cancer patients and represents the second leading cause of death [4]. Tumour-associated coagulopathy is essentially driven by the ability of tumour cells to activate the vascular endothelium. Endothelial cell activation (ECA) may transform the usually anti-coagulatory and anti-inflammatory endothelium into a pro-coagulatory, pro-inflammatory and strongly adhesive surface [5]. An activated tumour endothelium promotes binding of platelets and immune cells creating a pro-coagulatory and inflammatory tumour microenvironment [6, 7]. Previously, we presented evidence that the early binding of platelets to tumour endothelial cells through von Willebrand factor (vWF) was crucial for ECA because platelets release a plethora of activating compounds such as platelet derived growth factor, vascular endothelial growth factor-A (VEGF-A) and heparanase [5].

UBC patients suffer from hypercoagulation and previous studies showed that tissue factor (TF) was expressed on urothelial cancer cells and cancer derived microparticles [8, 9]. High TF expression is known to foster cancer progression and inversely correlates with disease-specific survival in patients with node-negative muscle-invasive UBC [10]. TF expression is also triggered by pro-inflammatory cytokines such as interleukin 1 (IL-1) or IL-6, connecting coagulation and inflammation. Previous research demonstrated an inverse correlation between IL-6 expression and UBC-specific survival [11, 12]. A key regulator of IL-6 expression is nuclear factor kappa-B (NF-kB), which is activated by pro-inflammatory cytokines such as IL-1 [13]. Constitutive NF-kB activation has been found in different cancers such as melanoma or nasopharyngeal carcinoma [14, 15]. In UBC, NF-kB function remains under debate; however, recent data identified a role in resistance to platin based chemotherapy as well as susceptibility to noxious agents contained in cigarette smoke [16–18]. IL-1 mediated NF-kB activation in endothelial cells triggers the surface exposure of vascular cell adhesion molecule-1 (VCAM-1) and intercellular cell adhesion molecule-1 (ICAM-1), ultimately facilitating the binding of blood flowing leukocytes and possibly enhancing diapedesis of tumour cells [19].

In the present study, we postulated that UBC cells had the ability to activate endothelial cells through inflammatory cytokines. Moreover, we hypothesized that the released cytokines promoted the generation of a pro-inflammatory and pro-coagulatory micromilieu, the recruitment of leukocytes and the loosening of the vascular barrier. Because of the heterogeneous nature of UBC cells, we compared the ability of different UCB cells to promote ECA. Finally, we aimed to verify our findings in biopsies of UCB patients by immune histology and transcriptome analysis.

## Methods

Additional information is given in the supplemental methods section.

### Cell culture

The human UBC cell lines were obtained from the European collection of authenticated cell cultures, RT4 (Catalogue No.: 91091914), RT112 (Catalogue No.: 85061106) and T24/83 (Catalogue No.: 85061107). The simian virus 40 large T antigen immortalized UROtsa cell line served as a model for the benign urothelium and were originally generated by Petzoldt et al. [20]. UROtsa cells were provided by Prof. Dr. Phillip Erben (University Hospital Mannheim, Germany). All cells were cultured in 75 cm^2^ flasks containing RPMI 1640 medium, enriched with 1% nonessential amino acids, 1% glutamine, 1% antibiotics (penicillin and streptomycin) and 10% foetal bovine serum (Boehringer Mannheim, Mannheim, Germany). The incubation was performed at 37 °C in a humid atmosphere of 5% carbon dioxide. Twice a week the media were changed with sub-cultivation at 80 to 90% of confluency. Every six month, cell lines were tested negative for Mycoplasma using the VenorGeM Classic Kit (Minerva Biolabs GmbH). Prior to the conducted research, cell line authenticity via identification of cell-specific single nucleotide polymorphisms was confirmed (Multiplexion GmbH, Heidelberg, Germany). Human umbilical vein endothelial cells (HUVECs) were freshly isolated from umbilical cords as previously described [21]. HUVECs were cultivated in culture medium composed of two-thirds M199 supplemented with 10% heat-inactivated foetal calf serum, 1% antibiotics (penicillin and streptomycin) and one third EGM-2 (Lonza, Basel, Switzerland). HUVECs were maintained in 25 cm^2^ flasks at 37 °C, 5% carbon dioxide and cultivated maximally up to the third passage.

### Generation of UBC cell derived supernatant (SN)

For standardized generation of SN, UBC cells were grown to confluence in a 75 cm^2^ flask. After removal of the culture medium, cells were thoroughly washed with HEPES-buffered Ringer’s solution (HBRS) that consisted of 140 mmol/L NaCl, 5 mmol/L KCl, 1 mmol/L MgCl_2_, 1 mmol/L CaCl_2_, 5 mmol/L glucose, and 10 mmol/L HEPES and had a pH of 7.4. After incubation with 10 ml RPMI 1640 without additives (starvation medium) for 24 h, the conditioned medium was harvested from the intact cell layer and centrifuged at 1800 g for 10 min to eliminate cell debris. Finally, the SNs were frozen at − 80 °C until experiments were performed.

### Stimulation of endothelial cells

For immunofluorescence staining, ECs were grown on gelatine-coated coverslips in standard culture 12-well plates. At confluency, culture medium was removed and cells were rinsed twice with pre-warmed HBRS. Subsequently, different stimuli (UBC cell SN alone or in combination with inhibitors (anti IL-1α 20 ng/ml (R&D systems, Wiesbaden, Germany); anti-IL-1β 30 ng/ml (R&D systems, Wiesbaden, Germany); anti-IL1ra 320 ng/ml (R&D systems, Wiesbaden, Germany)) were added to the cells at indicated concentrations. Pre-warmed starvation medium served as a control. The SN of endothelial cells were collected after 6 h of incubation at 37 °C, centrifuged at 300 g for 5 min to clear detritus, and kept at − 20 °C for later enzyme-linked immunosorbent assay (ELISA) analysis. The cells on coverslips were fixed and subjected to immunofluorescence staining. For qRT-PCR or flow cytometry, HUVECs were grown to confluence in standard 10 cm petri dishes or 25 cm^2^ flasks, then medium was removed, and cells were rinsed twice with pre-warmed HBRS. Subsequently, T24 cell SN or starvation medium (control) was added and incubated for 12 h.

### Immunohistochemistry (IHC)

Tissue samples of 105 bladder cancer patients were analysed for IL-1ra expression after obtaining approval by the local ethics committee (reference number 2007–030 N-MA). About one third of patients had undergone radical cystectomy for muscle invasive tumours while the rest was treated with transurethral resection. Approximately 60% of the patients suffered from a high grade tumour. The tissue was cut (4 μm thickness), placed on glass slides, and dried overnight at 36 °C. Subsequently, slides were deparaffinised and rehydrated, followed by a peroxidase block and washed several times in Tris-buffered saline. After steam heating with a target retrieval solution, slides were rinsed with ice-cold water. Slides were incubated overnight at 4 °C with a rabbit polyclonal anti-human IL-1ra antibody (Sigma-Aldrich, St. Louis, MO, USA) 1:200 in DAKO Real antibody diluent (DAKO, Glostrup, Denmark). After two washing steps in Tris-buffered saline, secondary EnVision HRP-labelled anti-rabbit antibody (DAKO, Glostrup, Denmark) was added for 40 min incubation at room temperature. After three more washing steps, visualization was performed with AEC + substrate chromogen (DAKO, Glostrup, Denmark) as indicated by the manufacturer. Counterstaining was performed with haematoxylin. Finally, coverslips were mounted with Faramount aqueous medium. Brightfield images of the samples were taken with an Observer Z.1 equipped with an EC-Plan-Neofluar 10x/NA0.3 objective and an AxiCam MRc camera (Zeiss, Jena, Germany). Image acquisition and analysis was performed with Zen software version 1.1.2.0 (Zeiss).

### Electric cell-substrate impedance sensing (ECIS)

We continuously measured the impedance of an endothelial cell monolayer as previously reported [22]. HUVECs, at a count of 10^5^, were grown to confluence in standard medium on planar gold-film electrodes deposited on the bottom of an 8-well electrode array (ECIS Cultureware 8W10E+, Applied BioPhysics Inc., Troy, NY, USA). Measurements were performed under standard cell culture conditions. Impedance was continuously measured using an ECIS 1600R instrument (Applied BioPhysics, Inc.) at a sampling frequency of 4 kHz, as this was proven to be the most sensitive frequency in assessing endothelial barrier function [23]. After 20 h, half of the endothelial cell culture medium was replaced by starvation medium as a control or by tumour cell SN with or without distinct inhibitors ((anti-IL1ra 320 ng/ml (R&D systems, Wiesbaden, Germany); anti-CXCL1 150 ng/ml (LSBio, Seattle, USA); anti-IL6 120 ng/ml (R&D systems, Wiesbaden, Germany); anti-IL8 50 ng/ml (R&D systems, Wiesbaden, Germany).

### Microfluidics

A laminar flow was induced in microfluidic devices that employed an air pressure based pump system (IBIDI GmbH, Munich, Germany) as previously described [5, 24]. Briefly, 0.5 × 10^6^ HUVECs were cultured on gelatine coated μ-slides 0.2 Luer (IBIDI GmbH, Munich, Germany) for 48 h under slight flow (1 dyn/cm^2^). Where indicated, slides were pre-incubated for 9 h with T24 cell SN or starvation medium as a control. Consecutively, each slide was perfused either with HBRS supplemented with 40% washed erythrocytes and 200,000 fluorescent platelets/μl or with hirudinated whole blood. Perfusion was performed with a shear stress of 6 dyn/cm^2^ for 15 min. Calcein green (Invitrogen, Darmstadt, Germany) labelled platelets bound to vWF strings, calcein blue labelled HUVECs (shown in white) were detected in real time using fluorescence microscopy equipped with a 20x objective (Observer.Z1, Zeiss, Jena, Germany). After the experiment, slides were fixated with 4% (v/v) paraformaldehyde (Electron Microscopy Science, Hatfield, USA) in HBRS. Antibodies directed against CD31 (mouse anti-human monoclonal antibody, Agilent Dako, dilution 1:150) as endothelial marker, CD45 (rat anti-human monoclonal antibody, abcam, dilution, 1:100) as leukocyte marker and von Willebrand factor (polyconal rabbit anti-human, Agilent Dako, dilution 1:200) as platelet marker were diluted in HBRS containing 1%BSA. Secondary antibodies conjugated to Alexa Fluor 488, 555 and 647 (ThermoFisher Scientific, Waltham, USA) were diluted in HBRS containing 1%BSA at dilutions of 1:200, 1:400 and 1:1000, respectively. DAPI was used to label nuclei. Slides were imaged with a 20x and 40x oil objective mounted to an Observer Z.1 (Zeiss). Data were processed with Zen software (1.1.2.0) and ImageJ (1.50c).

### Statistical analysis

Results were expressed as mean ± SD of at least three independent experiments. Statistical analysis was performed with GraphPad Prism software and significance was proven with an unpaired Student’s t-test or a Wilcoxon-Mann-Whitney-Test, as appropriate (* *P* ≤ 0.05; ** *P* ≤ 0.01).

## Results

### Cytokine release profile of UBC cells

**Fig. 1:**
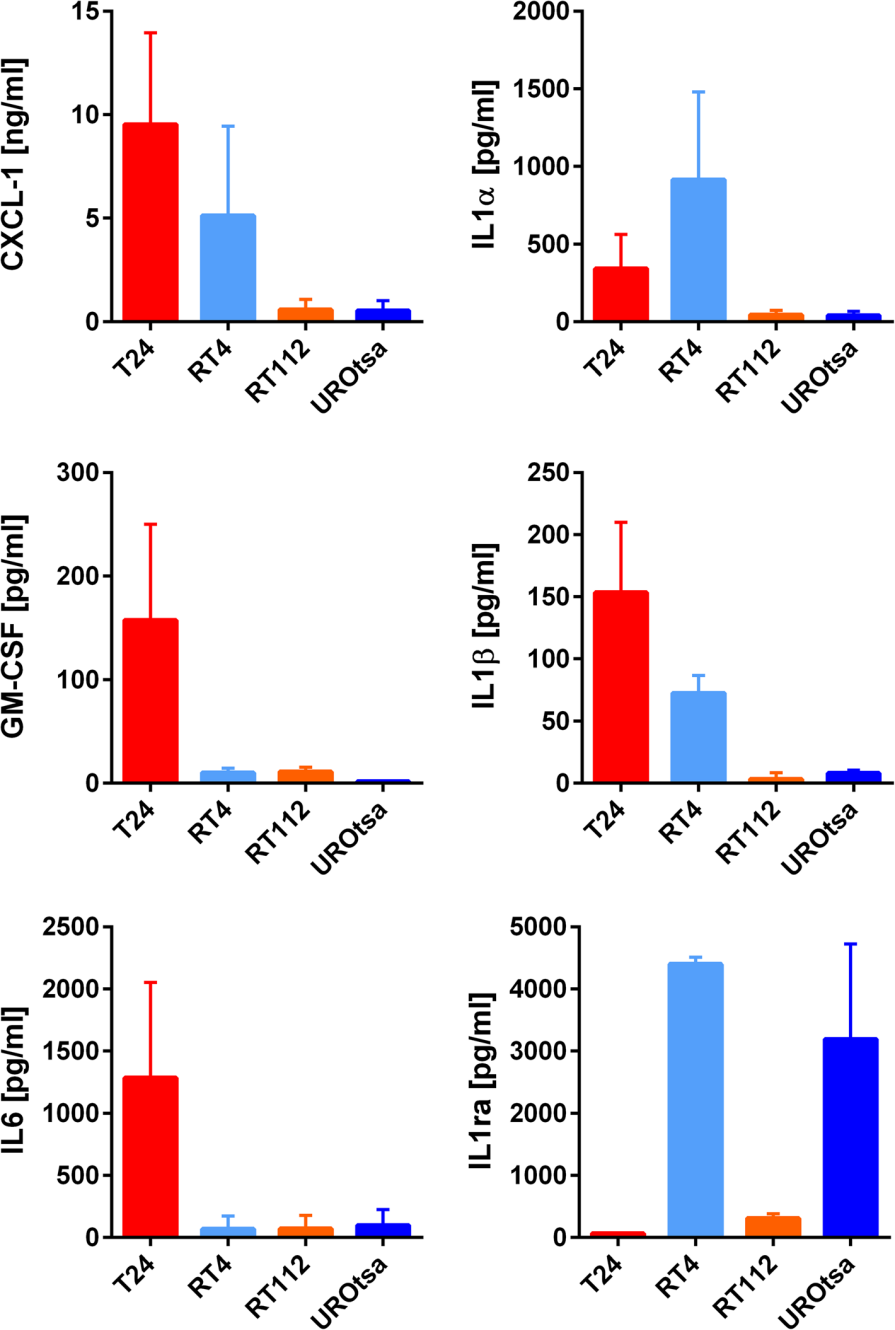
Cytokine release profile of distinct UBC cell lines. ELISA measurement of cytokine release by UBC cells after 24 h cultivation in starvation medium. Except for IL1α, cytokine release was highest in T24 cells. By contrast, secretion of inhibitory IL1ra was almost absent in T24 cells. IL: Interleukin; IL1ra: IL1 receptor antagonist; CXCL-1: C-X-C motif ligand-1; GM-CSF: Granulocyte-macrophage colony-stimulating factor; *n* = 3

Different concentrations of selected pro-inflammatory cytokines IL-6, IL-8, chemokine C-X-C motif ligand (CXCL)-1, IL-1α, IL-1β, IL-1 receptor antagonist (IL-1ra), Granulocyte-macrophage colony-stimulating factor (GM-CSF) were measured by ELISA, in the SN of different UBC cell lines (RT4, T24/83, RT112, UROtsa). The distinct UBC cells differed in release profiles. In comparison, T24 cells and, to a much lesser extent, RT4 cells, secreted the highest amounts of most of the assayed molecules. However, IL-1ra was almost absent in the SN of T24 cells, whereas high levels were detected in the SN of RT4 and UROtsa cells (Fig. 1).

### UBC cell mediated endothelial cell activation

**Fig. 2:**
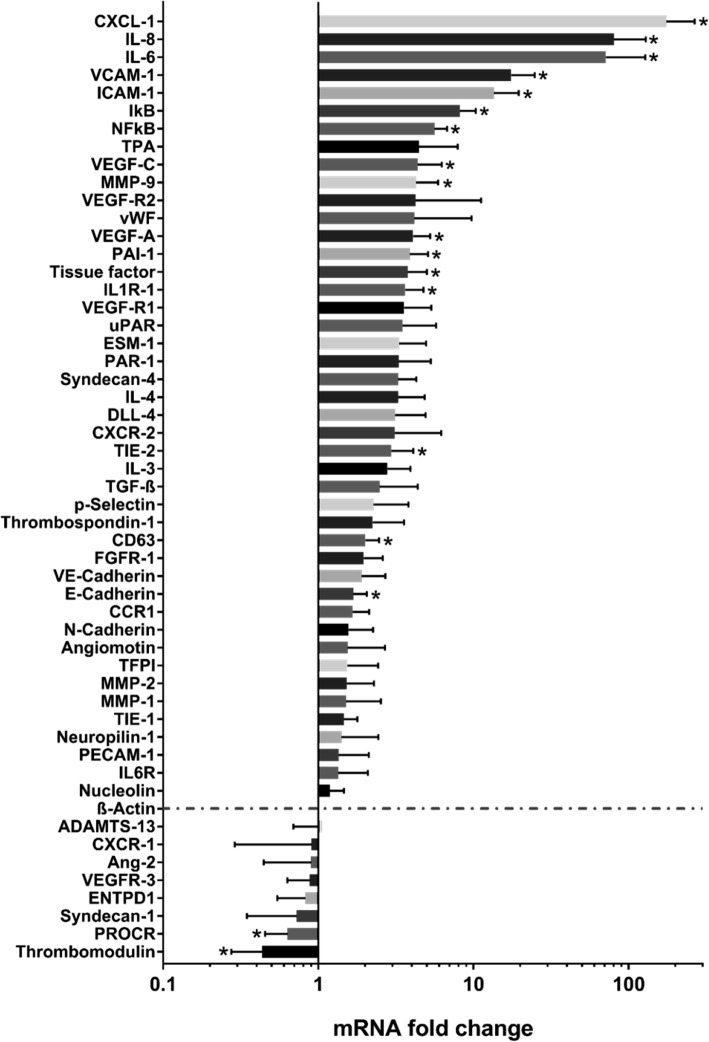
qRT-PCR gene profiling of HUVECs after 12 h exposure to tumour secretome. Genes promoting angiogenesis, immune cell recruitment, inflammation, cell adhesion, extracellular matrix remodelling and haemostasis (CXCL-1, IL-6, IL-8, VCAM-1, ICAM-1, VEGF-A, VEGF-C, MMP-9, TF, PAI-1) were up-regulated. Conversely, mRNA levels of genes inhibiting blood coagulation (thrombomodulin, PROCR) were reduced. Values were normalized to β-Actin (red dashed line). ICAM-1: Intercellular adhesion molecule 1, VCAM-1: vascular cell adhesion molecule 1, VEGF: Vascular Endothelial Growth Factor, MMP: Matrix metalloproteinase; *n* = 4 *P ≤ 0.05

The expression of 53 genes potentially involved in coagulation and inflammation was analysed in HUVECs after stimulation with SN of T24 cells. After 12 h exposure, we detected by qRT-PCR a significant up-regulation of genes promoting angiogenesis, immune cell recruitment, inflammation, lymphangiogenesis, cell adhesion and extracellular matrix remodelling (NF-kB, I-kB, CXCL-1, IL-6, IL-8, VCAM-1, ICAM-1, VEGF-A, VEGF-C, Matrix metalloproteinase-9 (MMP-9); Fig. 2). Moreover, the expressions of TF and Plasminogen activator inhibitor-1 (PAI-1) were elevated, whereas anti-coagulatory genes (thrombomodulin, Endothelial protein C receptor (PROCR)) were down-regulated. Interestingly, the increased transcription of the IL-1 receptor, IL1-R1, indicated an enhanced endothelial responsiveness towards IL-1. Transcription of pro-inflammatory NF-kB and its physiological inhibitor IκBα was concurrently enhanced, thus mRNA levels provided no definitive conclusion to which extent this pathway was activated. In line with our qRT-PCR results, flow cytometric analysis confirmed the up-regulation of TF, VCAM-1 and the down-regulation of thrombomodulin in HUVEC upon incubation with T24 cell SN (Supplemental Fig. 1).

**Fig. 3:**
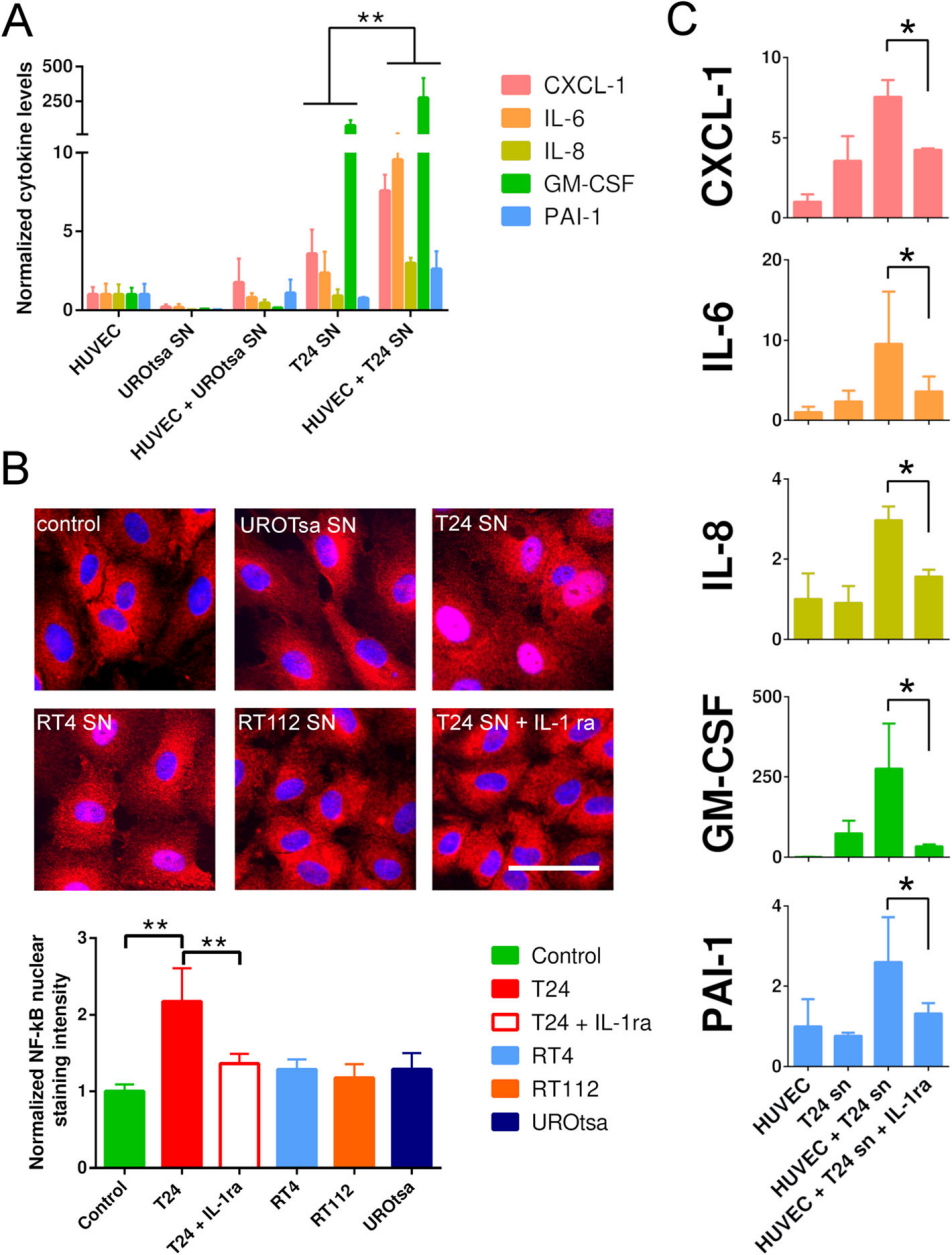
Pro-inflammatory activation of endothelial cells after stimulation with tumour cell SN. **a** After incubation with T24 cell SN, CXCL-1, IL-6, IL-8, GM-CSF and PAI-1 were significantly released from HUVECs (HUVECs + T24 cell SN). The SN of UROtsa was not able to stimulate HUVECs (HUVECs + UROtsa SN). HUVECs: baseline cytokine levels produced by HUVECs; T24 and UROTsa cell SN: baseline levels of cytokine produced by the tumour cells; (*n* = 8–12) ** *P* ≤ 0.01. **b** Incubation of HUVECs for 6 h with T24 cell SN promoted the trans-localization of NF-kB (red) into the nucleus (blue) of the endothelial cells. Treatment of HUVECs with the SN of RT112 and UROtsa cells had no effect. RT4 cell SN induced a weak trans-localization of NF-kB. Addition of IL-1ra blocked NF-kB translocation upon treatment with T24 cell SN. The bar diagram shows the fluorescence intensity of nuclear NF-kB. Scale bar corresponds to 50 μm; (*n* = 10 images) ** *P* ≤ 0.01. **c** IL-1ra inhibited the T24 cell SN induced release of the indicated pro-inflammatory cytokines from HUVECs. Cytokine levels were shown in pg/ml; (*n* = 3) * *P* ≤ 0.05

Many of the cytokines that were up-regulated in HUVECs after stimulation with T24 cell SN could be detected in the T24 secretome in abundant concentrations. This indicated an amplification of the tumour cell cytokine signature by the activated vascular endothelium. To further validate this finding, we quantified selected endothelial derived cytokines after incubation with T24 cell SN by ELISA. In comparison to the T24 cell SN, we found a significant increase of the pro-inflammatory, pro-angiogenic, immune-stimulatory and pro-thrombotic molecules CXCL-1 (fold increase: 2.1, *P* = 8.5 × 10^− 5^, 95% CI: 6.6–12.4 vs 18.7–21.8 μg/ml), IL-6 (fold increase: 4.1, *P* = 0.005; 95% CI: 1.2–1.7 vs 2.9–7.4 μg/ml), IL-8 (fold increase: 2.1, *P* = 1.02 × 10^− 8^, 95% CI: 3.0–5.9 vs 13.5–15.6 μg/ml), GM-CSF (fold increase: 3.7, *P* = 0.008, 95% CI: 90–225 vs 378–792 pg/ml) and PAI-1 (fold increase: 2.9, *P* = 0.01, 95% CI: 11.3–13.3 vs 25.0–45.2 pg/ml) in the SN of the endothelial cells (Fig. 3a). After treatment of HUVECs with UROtsa cell SN, none of the measured cytokines was found to be elevated.

### Endothelial cell activation is IL-1 dependent

IL-1α and IL-1β prominently released by T24 cells (Fig. 1) are potent activators of the NF-kB pathway [25]. To confirm our hypothesis, we stained NF-kB in HUVEC monolayers by immunofluorescence after exposure to UBC cell conditioned medium. In contrast to the SN of UROtsa and RT112 cells, SN of T24 cells caused a strong nuclear translocation of NF-kB indicating an activation of this pathway. RT4 cell SN had only a weak effect on NF-kB translocation probably reflecting the high amount of IL-1ra in the SN of RT4 cells (Fig. 3b). Indeed, the addition of human recombinant IL-1ra to the T24 cell SN markedly reduced NF-kB translocation. This further underline the idea of an IL-1 mediated activation of the endothelium.

Since blockage of the IL-1 pathway by IL-1ra reduced NF-kB activation, we investigated whether the T24 cell SN induced release of endothelial cytokines was also IL-1 dependent. In line with the NF-kB activation, co-treatment with IL-1ra abolished the T24 cell SN induced release of CXCL-1, IL-6, IL-8, GM-CSF, PAI-1 (Fig. 3c). Thus, IL-1 seems primordial in creating a tumour induced pro-thrombotic, pro-inflammatory and pro-angiogenic intravascular microenvironment.

### UBC cell promoted endothelial barrier breakdown

**Fig. 4:**
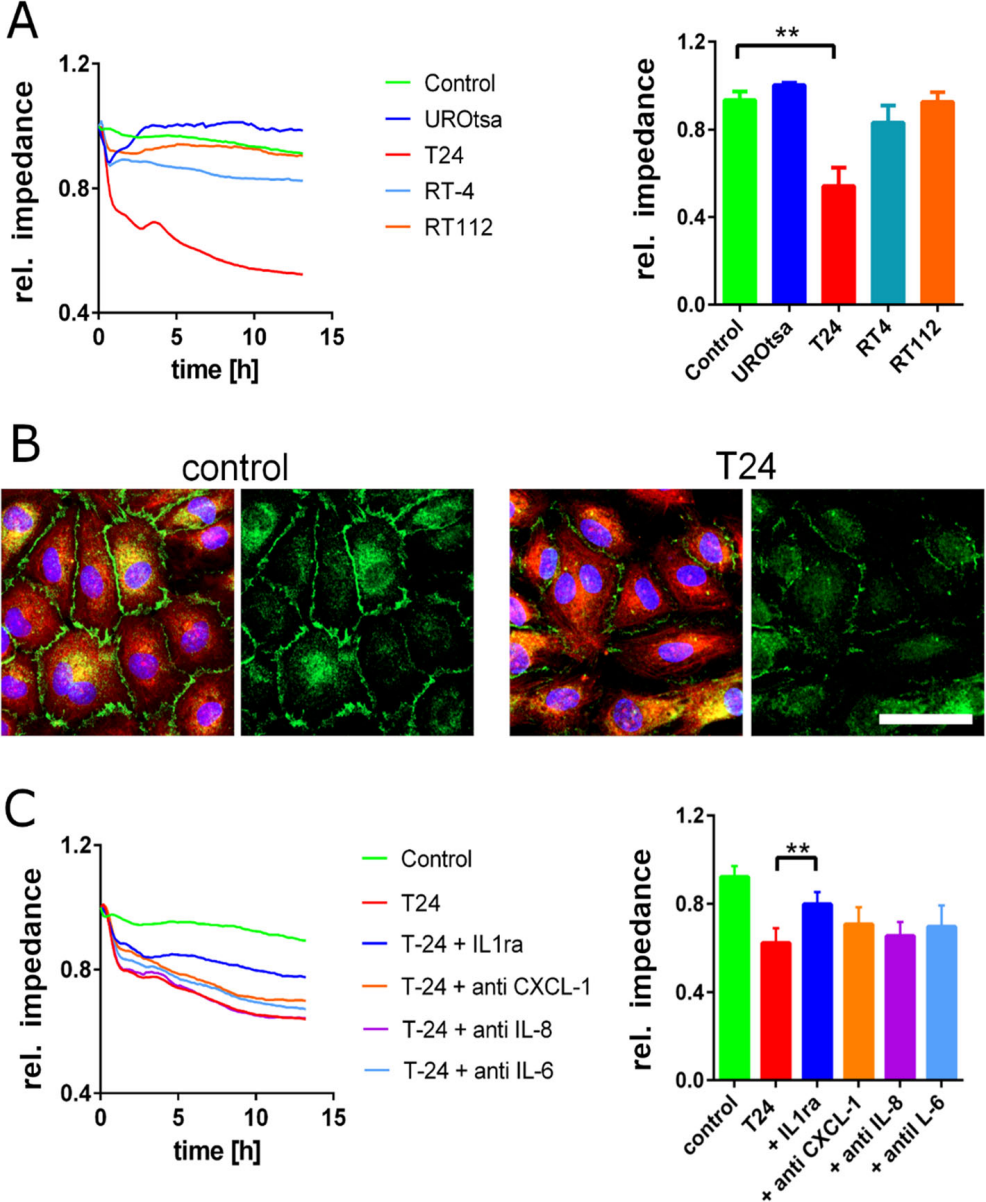
Impact of UBC cell SN on the integrity of the endothelial cell layer. **a** HUVECs grown on gelatine coated ECIS electrodes were treated with starvation medium (control) or UBC cell SN. The impedance was measured continuously up to 12 h after treatment. T24 cell SN led to marked breakdown of the endothelial impedance. RT4 cell SN had a lower impact whereas RT112 cell SN, UROtsa cell SN or starvation medium (control) did not cause significant alterations after 12 h; *n* = 5; ** *P* ≤ 0.01. **b** HUVEC cells were incubated for 12 h with T24 cell SN (T24) or starvation medium (control) and then analysed by immunofluorescence staining of β-Actin (red) and CD31/PECAM-1 (green). In comparison to the control (left), treatment with T24 cell SN (right) induced the disintegration of adherent junctions (CD31/PECAM-1) indicating a weakening of the endothelial barrier. **c** Inhibition of cytokine signalling partially conserved endothelial barrier function. Antibodies against IL-6, CXCL-1 slightly mitigated impedance breakdown. In contrast, IL-1ra strongly attenuated tumour mediated endothelial dysfunction, whereas IL-8 neutralization had no impact; n = 4; ** *P* ≤ 0.01

To assess the role of bladder cancer-mediated ECA on endothelial cell integrity, we measure the impedance of a HUVEC monolayer non-invasively by ECIS. As shown in Fig. 4a, T24 cell SN caused a marked impedance breakdown (fold decrease: 1.5, *P* = 1.5 × 10^− 7^, 95% CI 0.89–0.95 vs 0.58–0.66), whereas RT112 and UROtsa cell SN had almost no effect. Loss of adherent junctions in the HUVEC monolayer confirmed the endothelial barrier disruptive effect of the T24 cell SN. Furthermore, the β-actin cytoskeleton became more pronounced indicating the formation of stress fibres (Fig. 4b).

Consistent with the results shown in Fig. 3, blockage of the IL-1 receptor by IL-1ra significantly attenuated T24 cell SN induced breakdown of the endothelial barrier (fold increase: 1.3, *P* = 0.085, 95% CI: 0.56–0.66 vs 0.75–0.85). Inhibition of IL-6 and CXCL-1 counteracted the impedance decrease only slightly. Neutralization of IL-8 had no impact (Fig. 4c).

### UBC cell induced platelet binding and leukocyte recruitment

**Fig. 5:**
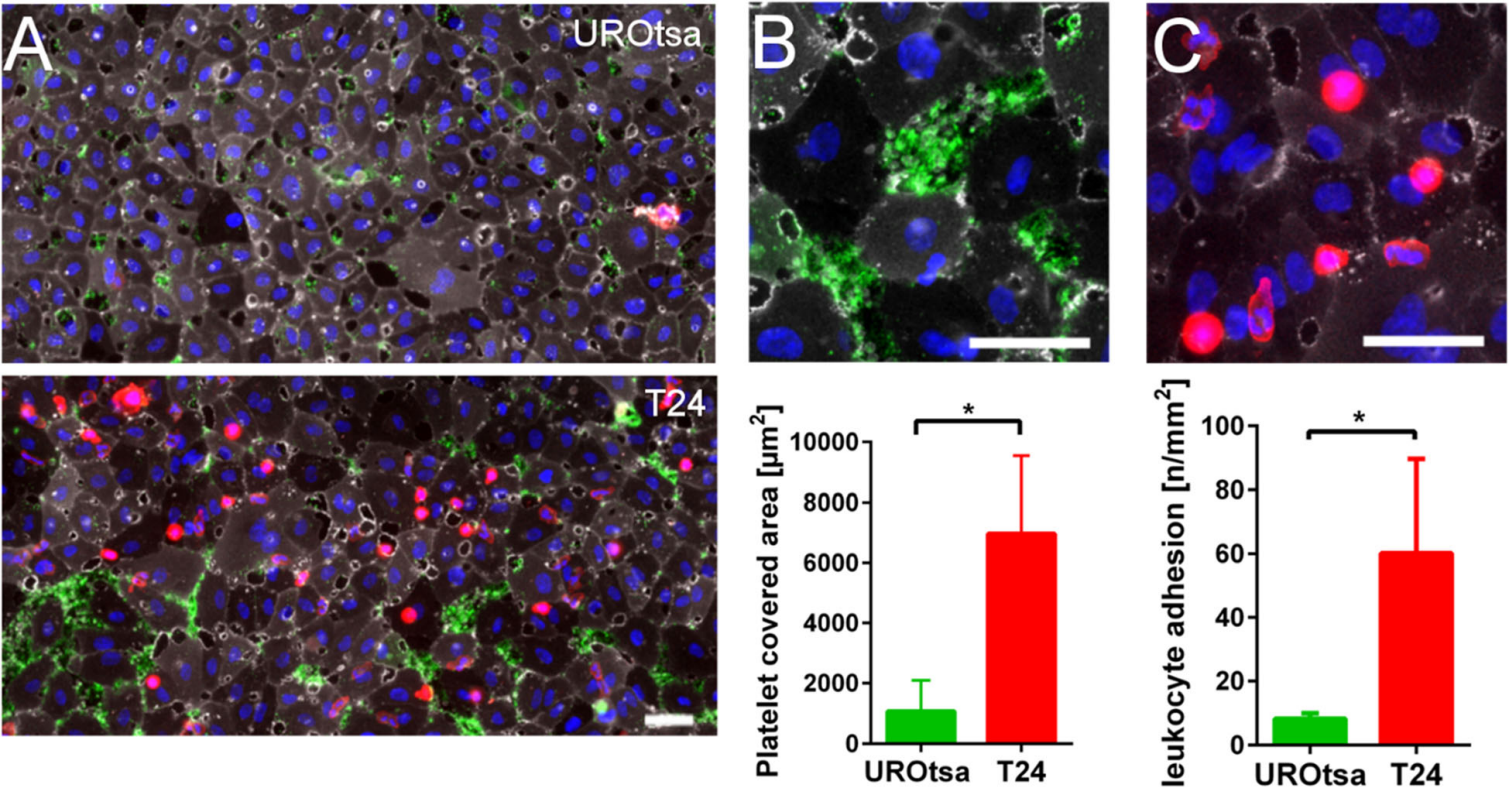
Microfluidic experiments indicated the pro-inflammatory and pro-coagulatory activation of endothelial cells by tumour cell SN. HUVECs were perfused at 6 dyn/cm^2^ with hirudinated whole blood for 15 min. **a**, **b** In comparison to the SN of UROtsa cells, pre-treatment of HUVECs with T24 cell SN for 6 h promoted platelet binding (green) to gaps between the endothelial cells (white). CD31 was used as endothelial marker, vWF was used to identify platelets. (Additional experiments performed with washed and fluorescent platelets are shown in the data supplement.) **b** Magnified region of platelets bound to HUVECs treated withT24 cell SN. Quantitative analysis of the platelet covered area is shown as bar diagram. n = 3; * *P* ≤ 0.05 **a**, **c** Pre-treatment of HUVECs with T24 cell SN mediated the recruitment of leukocytes (red). **c** Magnified region of leukocytes attached to HUVECs treated withT24 cell SN. CD45 was used as leukocyte marker. The number of recruited leukocytes is shown as bar diagram; n = 3; * *P* ≤ 0.05. **a**-**c** Nuclei (blue) were stained with DAPI, scale bars correspond to 50 μm

To further investigate the consequences of tumour-induced alterations of the endothelial surface, we applied a microfluidic artificial vessel system. Our microfluidic setup mimics pathophysiological blood flow conditions and enables the real-time visualisation of platelet and leukocyte binding. HUVEC coated microfluidic channels were perfused with a mixture of platelets and 40% haematocrit or with whole hirudinated blood at a shear stress of 6 dyn/cm^2^. We measured platelet binding (platelet covered area) as an indicator of pro-thrombotic endothelial cell activation. Previously, we showed that platelets could be trapped at the vessel wall by ultralarge vWF fibres released from activated endothelial cells through a VEGF-A mediated signalling [26]. However, our previous research indicated that the SN of T24 cells was unable to instantaneously mediate the release of vWF from HUVECs [26]. Here, we measured the binding of platelets upon prolonged pre-treatment of the HUVECs (6 h) with T24 cell SN (Fig. 5a, b and Supplemental Fig. 2A, B). Independent from ultralarge vWF fibres, platelets accumulated in between HUVECs, which had lost their cell-cell contacts. This suggests that increased platelet binding was mediated by subendothelial vWF (fold increase: 6.4, *P* = 0.003, 95% CI: 304–1857 vs 5854–8075). As shown in Fig. 5a, c and Supplemental Video 1, binding of platelets was accompanied by an increased leukocyte recruitment (fold increase: 7.3, *P* = 0.04, 95% CI: 6.6–9.9 vs 32.9–87.4). This may at least partially reflect the elevated expression of ICAM-1 and VCAM-1 by T24 stimulated endothelial cells (Fig. 2). Co-perfusion but not pre-treatment of the endothelial layer with T24 cell SN promoted further platelet binding (Supplemental Fig. 2C, D) indicating a tumour SN mediated activation of platelets. Indeed, light transmission aggregometry confirmed that the SN of T24 cells promoted platelet aggregation (Supplemental Fig. 2E**)**. Interestingly, IL-1ra quenched the T24 cell SN induced platelet aggregation only slightly and the stimulation of platelets with recombinant human IL-1β promoted only a weak aggregation of platelets. Therefore, IL-1 appears to be only marginally involved in direct platelet activation suggesting the contribution of other T24 cell secreted factors.

### IL-1 and IL-1ra expression in bladder cancer tissues

**Fig. 6:**
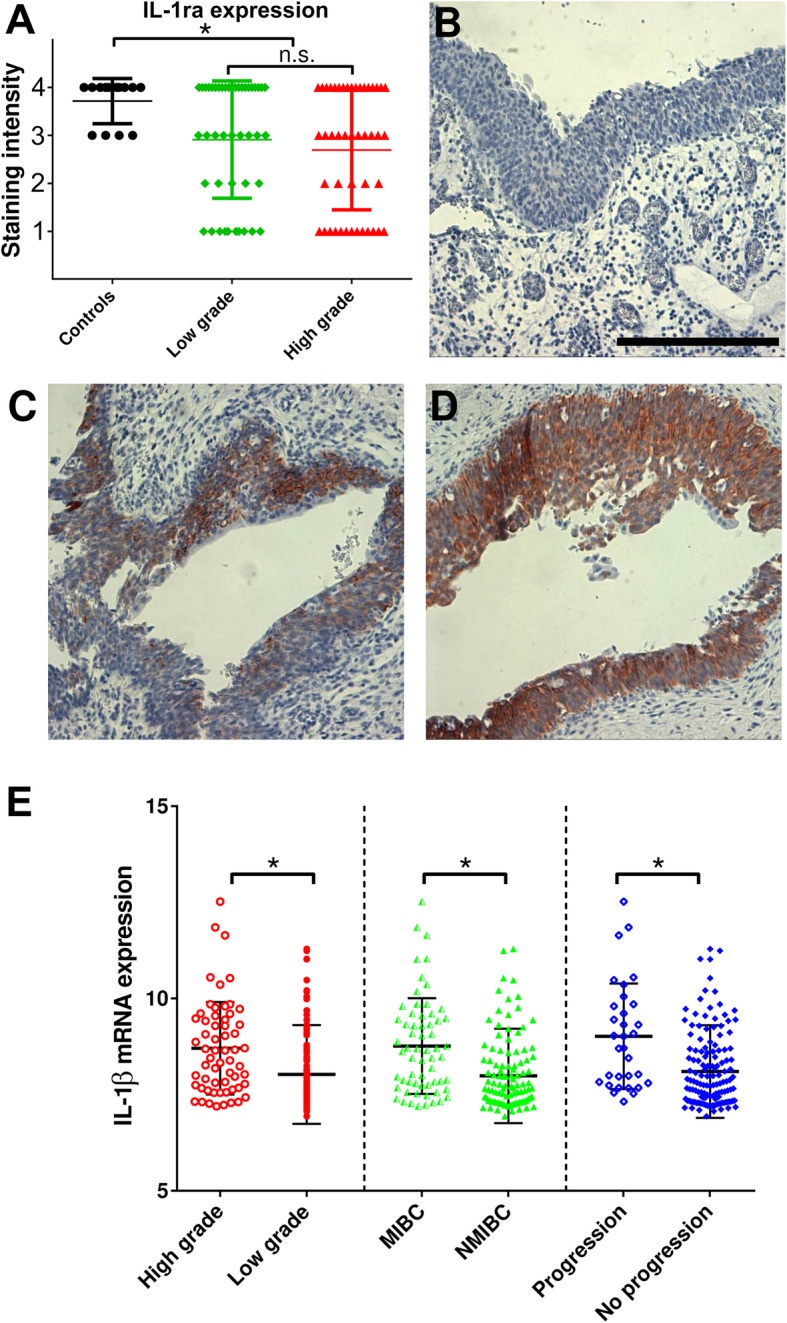
Impact of IL-1 on bladder cancer progression. **a** IL-1ra staining intensity in a cohort of 105 patients with UBC measured semiquantitatively by IHC. Controls (benign urothelium) showed a higher IL1-ra expression than in patients with cancer. No significant difference was found between low grade and high grade tumours. The staining intensity score was defined as follows: no staining = 1, low intensity = 2, moderate intensity = 3 and high intensity = 4. * *P* ≤ 0.05. n.s. = not significant. Representative IHC images are shown in **b**-**c**. Scale bar corresponds to 500 μm. **b** IHC very low staining intensity (score = 1). **c** Intermediate staining intensity (score = 3). **d** High staining intensity (score = 4). **e** High IL-1β mRNA levels were linked to adverse clinicopathological features high grade disease, muscle invasiveness and tumour progression. MIBC: muscle invasive bladder cancer, NMIBC: non-muscle invasive bladder cancer; * *P* ≤ 0.05

In tissue biopsies from a cohort of 105 patients with UBC, expression of IL-1ra was quantified by IHC. Bladder tumours showed significantly lower IL-1ra staining intensity compared to normal urothelium (Fig. 6a-d). Secretion of IL-1 and loss of antagonistic IL-1ra characterized T24 cells (Fig. 1). To investigate whether elevated IL-1β mRNA expression in UBC patients was linked to clinicopathological features, we additionally analysed two publicly available datasets. Gene expression was correlated with grading, muscle invasiveness and disease progression. IL-1α and IL-1ra mRNA levels showed no consistent correlation with clinicopathological features (data not shown). However, high IL-1β expression was measured in patients with adverse disease characteristics (Fig. 6e).

## Discussion

Considerable progress in cancer therapy has been achieved over the last years. However, survival rates of patients with metastatic UBC are still very limited [27]. For the colonization of distant sites, cancer cells must leave the primary tumour and enter and exit the blood circulation. The process of intravasation and extravasation is termed trans-endothelial migration and the vascular endothelial layer represents a critical barrier for tumour cells [28]. In contrast to the usually leaky and dysfunctional tumour neo-vessels, the endothelial surface in distant organs is intact. Therefore, it is highly probable that trans-endothelial migration is an active process fostered by cytokines, growth factors and vascular permeability regulators. In response to these environmental stimuli, endothelial cells adapt to the new conditions and undergo activation (ECA), which in turn creates pro-inflammatory and pro-coagulatory intravascular conditions. Therefore, ECA can enable multiple heterotypic adhesive interactions between the endothelium and blood flowing tumour cells, platelets and leucocytes which further mediates tumour cell extravasation and metastasis [29–31].

In this study, we investigated the inflammation–coagulation axis of UBC-induced ECA and its role for local invasion and tumour progression. Although the pathophysiological contribution of endothelial cells in UBC is still unclear, previous data suggest their central role in immune cell recruitment and tumour progression [32].

UBC cells produce a variety of pro-angiogenic, pro-inflammatory cytokines, such as IL-1, CXCL-1 or IL-6, which accelerate tumour growth and metastasis formation in an auto- and paracrine manner [11, 33, 34]. The immunogenic potential of the cells used in our study differs significantly. We found that T24 cells released the highest amount of IL-1β, IL-6, CXCL-1 and GM-CSF. RT4 cells released higher levels of IL-1α and its antagonist IL-1ra. RT112 and UROtsa cells showed comparable less cytokine production. Although the UBC cell secretome has previously been studied and partially correlated with adverse clinicopathological features [11, 34–39], the functional impact of the secretome on ECA received considerably less attention. To further characterize ECA, we developed a RT-qPCR panel comprising 53 genes involved in endothelial homeostasis, intravascular coagulation, regulation of inflammation, chemotaxis and cell adhesion. Our RT-qPCR results showed that tumour-induced transcriptional changes promoted a pro-inflammatory, pro-coagulatory and cell adhesive endothelial phenotype. Interestingly, we measured the increased expression of the IL-1 receptor IL1R-1 suggesting that endothelial cells may increase their sensitivity towards IL-1. It has been shown that IL-1 can directly be produced by inflamed endothelial cells [40]. Therefore a positive feedback regulation that fuel further endothelial activation appears to be likely.

Next to IL-1, T24 cell SN activated HUVECs express high levels of CXCL-1, IL-6 and IL-8. Of note, IL-1 can induce the expression of IL-6 and IL-8 in HUVECs in an autocrine manner [40]. The elevated production of CXCL-1 and IL-8, both potent leukocyte attracting and activating molecules, is in line with the endothelial recruitment of CD45^+^ cells in our microfluidic experiments. In the context of cancer, CXCL-1, IL-8 and IL-6 were known to tune the tumour microenvironment. However, our experiments suggest that neither CXCL-1, IL-6 nor IL-8 were by themselves strong activators of HUVECs. In vivo, CXCL-1 was shown to collaborate with IL-6 to activate endothelial cells and to promote angiogenesis in bladder cancer [41]. Whereas endothelial cells express the CXCL-1 receptor CXCR2 [42], they lack IL-6 receptor expression and they were only able to respond to IL-6 in the presence of soluble IL-6 receptor [43]. Within the tumour microenvironment, this trans-signalling pathway is likely to further promote endothelial cell activation and tumour progression [43, 44]. Also IL-8 has been linked to endothelial cell activation and angiogenesis in tumours [45]. Therefore, further research is needed to understand the complex cross talk between UBC cell derived cytokines and the (tumour) endothelium. Our data suggest that amplification of the tumour cell related cytokine signature by endothelial cells can promote the development of a tumour supportive microenvironment. Interestingly, a similar tumour promoting feedback loop in UBC has been described for tumour infiltrating fibroblasts [31].

Conversely, anti-coagulatory molecules such as thrombomodulin (TM) or PROCR were down-regulated in HUVECs after stimulation with T24 cell SN. Our findings are in line with previous studies showing a correlation between high levels of pro-coagulatory TF and a reduction in disease-specific survival in patients with pN0 muscle-invasive UBC [10]. Furthermore, decreased expression of TM in UBC may predict aggressive tumour growth and advanced clinical stage [46].

Because IL-1 is a potent NF-kB activator, we postulated that the IL-1 - NF-kB axis could be involved in UBC triggered ECA. We found that T24 cell SN induced NF-kB nuclear translocation in HUVECs was IL-1 dependent. In accordance with our hypothesis, members of the NF-κB family have been reported to promote urinary cancer of the bladder via increasing angiogenesis, resistance against cisplatin and enhanced epithelial mesenchymal transition [18, 47, 48].

Next, we were interested in functional changes of the endothelial barrier after exposure to the SN of UBC cell. We measured a strong breakdown of the endothelial layer integrity after exposure to T24 cell SN. To validate our theory that the UBC cell – endothelial cell interaction was mediated by IL-1, we blocked the potential signalling of different cytokines. In line with our assumption, IL-1ra strongly attenuated the disruption of the endothelial monolayer.

To evaluate the functional relevance of the IL-1 mediated ECA, we used an artificial blood vessel system mimicking the pathophysiological conditions of the tumour vasculature [5]. The tumour induced ECA generates a “sticky” vascular surface permitting an increased leukocyte and platelet binding under flow conditions. In UCB, the tumour microenvironment was shown to impact tumour progression and to induce recruitment of pro-tumorigenic regulatory T cells [35, 49]. Increased leukocyte binding to the tumour cell activated endothelium might therefore represent one crucial step of leukocyte attraction. Stimulation with T24 cell SN also promoted platelet binding and aggregation. Platelets are key players of the intravascular homeostasis and it is generally conceived that platelets may contribute to tumour cell dissemination via shielding circulating tumour cells from immunological recognition as well as facilitating tumour cell extravasation [31, 50].

To estimate the in vivo relevance of the IL-1 mediated ECA, we analysed biopsies of bladder cancer patients by IHC and the bladder cancer transcriptome in publicly available data sets. In concordance with one of our previous studies, IHC staining of tumour tissue from UCB patients revealed a significantly weaker IL-1ra expression when compared to healthy tissue samples [51]. Similarly, adverse clinicopathological features correlated with high IL-1β mRNA tissue levels in the UCB transcriptome. The IL-1 superfamily has 11 structurally comparable members, but IL-1α, IL-1β and IL-1ra are by far the best understood [52]. These cytokines constitute an essential part of the inflammatory response of the body against infection. In the last years, synergistic effects between inflammation and cancer invasion have come into focus and IL-1 might be a crucial player therein [53]. To date, the role of IL-1 in bladder cancer progression is not fully explored. Several aspects have so far been described, such as IL-1β induced cisplatin-resistance by up-regulation of Aldo-keto reductase 1C1 [54], IL-1 dependent intra-tumoural androgen receptor signalling, T-cell attraction or stimulation and recruitment of tumour-associated fibroblasts [33]. Furthermore, polymorphisms in the IL-1ra gene were associated with recurrence after Bacillus Calmette-Guerin immunotherapy and susceptibility to bladder cancer [55, 56].

## Conclusions

The present study demonstrated multiple tumour-induced alterations of the vascular endothelium in UBC that were previously not described in this entity. The discovered UBC-induced inflammatory ECA appears to be required for immune cell recruitment and disruption of the endothelial barrier that may further support tumour cell metastasis. We showed that secretion of UBC-derived IL-1 was highly relevant for the mutual interactions between tumour cells and the vascular endothelium. This crosstalk creates a pro-coagulatory, pro-inflammatory and pro-adhesive intravascular micromilieu and presents a possible target for anticancer therapies beyond classical chemotherapy or immunotherapy. Pharmacological inhibition of IL-1 receptor by anakinra attenuated bladder dysfunction in a murine haemorrhagic cystitis model [57]. Therefore, anakinra or other inhibitors of the IL-1 signalling should be considered as therapeutic option in bladder cancer to reconstitute vascular homeostasis and to reduce metastasis.

## Supplementary Information

**Additional file 1.** Supplemental Methods.

**Additional file 2: Fig. S1.** FACS analysis of HUVEC surface molecules after stimulation with T24 SN. **Fig. S2.** Platelet binding to T24 cell SN activated endothelial cells.

**Additional file 3: Video S1.** Live reflection interference contrast microscopy of HUVECs perfused with whole hirudinated blood.

## Data Availability

The datasets used and/or analysed during the current study are available from the corresponding author on reasonable request.

## Abbreviations

IL: Interleukin
IL-1ra: Interleukin 1 receptor antagonist
UBC: Urothelial bladder cancer
ECA: Endothelial cell activation
vWF: Von Willebrand factor
VEGF-A: Vascular endothelial growth factor A
TF: Tissue factor
ADAMTS13: A disintegrin and metalloproteinase with a thrombospondin type 1 motif, member 13
NF-kB: Nuclear factor kappa-B
VCAM-1: Vascular cell adhesion molecule-1
ICAM-1: Intercellular cell adhesion molecule-1
HUVEC: Human umbilical vein endothelial cells
SN: Supernatant
HBRS: HEPES-buffered Ringer’s solution
CXCL: Chemokine (C-X-C motif) ligand
GM-CSF: Granulocyte-macrophage colony-stimulating factor
PAI-1: Plasminogen activator inhibitor-1
TM: Thrombomodulin
ECIS: Electrical cell-substrate impedance sensing
MMP-9: Matrix metalloprotease 9
I-kB: NF-kB inhibitor
PROCR: Endothelial protein C receptor
IHC: Immunohistochemistry
CI: Confidence interval
