# Supplementary material for: Bladder cancer-derived interleukin-1 converts the vascular endothelium into a pro-inflammatory and pro-coagulatory surface
Source: BMC Cancer. 2020 Dec 2;20:1178. doi: 10.1186/s12885-020-07548-z (PMC7709388; doi:10.1186/s12885-020-07548-z)
Supplement: Supplementary file 1 — Additional file 1. Supplemental Methods. [file 12885_2020_7548_MOESM1_ESM.pdf]

Bladder cancer-derived interleukin-1 converts the vascular endothelium into a pro-inflammatory and pro-coagulatory surface

A. John[1], C. Günes[1], C. Bolenz[1], S. Vidal-y-Sy[2], A. T. Bauer[2], S.W. Schneider[2], C. Gorzelanny[2*]

[1] Department of Urology, University of Ulm, Ulm, Germany

[2] Department of Dermatology, University Medical Center Hamburg-Eppendorf, Hamburg, Germany

* Corresponding author: Christian Gorzelanny
Department of Dermatology,
University Medical Center Hamburg-Eppendorf,
Martinistraße 52, 20246 Hamburg, Germany
E-Mail: c.gorzelanny@uke.de
Tel.: +49 40 7410 58976
Fax: +49 40 7410 52655

Supplemental Methods

Enzyme-linked immunosorbent assay (ELISA)

The IL-6, chemokine C-X-C motif ligand-1 (CXCL-1), Granulocyte-macrophage colony-stimulating factor (GM-CSF), IL-1α, IL-1β, IL-1ra and Plasminogen activator inhibitor-1 (PAI-1) levels were quantified using the human DuoSet ELISA kit (R&D Systems, Wiesbaden, Germany) according to the manufacturer's instructions.

Immunofluorescence staining of endothelial cells

Morphological structures of cultivated endothelial cells were visualized using the indirect immunofluorescence staining technique. After stimulation with tumour SN with or w/o inhibitors [anti-IL1α 20 ng/ml (R&D systems, Wiesbaden, Germany); anti-IL1β 30ng/ml (R&D systems, Wiesbaden, Germany); anti-IL1ra 320 ng/ml (R&D systems, Wiesbaden, Germany)] for 6 hours, endothelial cells on coverslips were fixed in ice-cold methanol for 30min, washed with HBRS, and blocked in 2% bovine serum albumin in an incubation buffer (0.1% bovine serum albumin and 0.3% Triton X-100 in HBRS) for 1 hour at room temperature (RT). For detection, the mouse anti human NF-kB antibodies (Santa Cruz Biotechnology, Heidelberg, Germany) at a dilution of 1:200 and Alexa Fluor 555 conjugated goat anti-mouse (ThermoFisher Scientific, Waltham, USA) at a dilution of 1:400 were used in incubation buffer for 1 hour at RT. Nuclei were stained with 4,6 diamidino-2-phenylindole (DAPI) diluted 1:1000 in PBS for 10min. Finally, coverslips were embedded with Mowiol–glycol solution with freshly added 50 mg/ml of 1,4-diazabicyclo[2.2.2]octane (MilliporeSigma, Merck KGaA, Darmstadt, Germany). Fluorescence microscopy was performed using an Observer Z.1 (Zeiss, Jena, Germany). The microscope was equipped with a AxioCam MRm camera, filter sets to detect blue, green and red fluorescence (38HE, 43HE and 49) and following objectives: LD Plan-Neofluar 20x/NA 0.4; EC Plan-Neofluar 40x/NA0.75; EC Plan-Neofluar oil 40x/NA1.3. Fluorescence light with defined wavelengths (365 nm, 477 nm and 555 nm)

was generated by light-emitting diodes. Zen software (1.1.2.0) and ImageJ (1.50c) was used for image acquisition and processing.

RNA preparation and qRT-PCR

After 12h incubation with tumour SN, total RNA from HUVECs was extracted using the RNeasy mini kit (Qiagen, Hilden, Germany) and cDNA was synthesized from 1µg of total RNA per sample with the Reverse Transcription System from Promega according to the manufacturers' protocols. To quantify mRNA transcript levels, quantitative real-time polymerase chain reaction (qRT-PCR) was performed applying the GoTaq® qPCR Master Mix (Promega, Heidelberg, Germany). Following primers were used: **ADAMTS13**: 5'-ACA GGC CGT GTC TTC TTA CTT-3' and 5'-GGG TCC CGA AGC AGT TCT G-3'; **angiomotin**: 5'-TGT CAC TGG AGT CAC CAA CCA-3' and 5'-GCA ATG AGA TGT CCT GCG CT-3'; **angiopoietin (ang) 2**: 5'-CCT GTT GAA CCA AAC AGC GG-3' and 5'-AAC AGT GGG GTC CTT AGC TG-3'; **β-actin**: 5′-AGA AAA TCT GGC ACC ACA CC-3′ and 5′-CCA TCT CTT GCT CGA AGT CC-3′; **C-C chemokine receptor type (CCR) 1**: 5'-TTC GTT GGT GAG AGG TTC CG-3' and 5'-ACC CAG CAG AGA GTT CAT GC-3';**CD63**: 5'-TCC TCC TGC TGG CCT TTT G-3' and 5'-AAT CTG CCT GCA TCC TGT CC-3'; **C-X-C motif chemokine ligand (CXCL) 1**: 5'-GGG AAT TCA CCC CAA GAA CAT C-3' and 5'-GGA TGC AGG ATT GAG GCA AGC-3'; **C-X-C motif chemokine receptor (CXCR) 2**: 5'-AAA CTC CCT CGT GAT GCT GG-3' and 5'-CAG GCT GGG CTA ACA TTG GA-3'; **CXCR1**: 5'-GCT ATG GAT TCA CCC TGC GT-3' and 5'-GAT GAC GTG CCA AGA ACT CC-3'; **delta-like (DLL) 4**: 5'-CAG CAC TCC CTG GCA ATG TA-3' and 5'-CAC AGT AGG TGC CCG TGA AT-3'; **E-cadherin**: 5'-CTT TGA CGC CGA GAG CTA CA-3' and 5'-TGG ATT CCA GAA ACG GAG GC-3'; **ectonucleoside triphosphate diphosphohydrolase (ENTPD) 1**: 5'-AGG TGC CTA TGG CTG GAT TAC and 5'-CCA AAG CTC CAA AGG TTT CCT-3'; **endothelial cell-specific molecule 1 (ESM1)**: 5'-ACT TGC TAC CGC ACA GTC TC-3' and 5'-GCA GGT CTC TCT GCA ATC CA-3'; **endothelial protein C receptor (PROCR)**: 5'-CCT ACA ACC GCA CTC

GGT ATG-3' and 5'-CGC GGA AAT ATG TTT CTG CAC A-3'; **fibroblast growth factor receptor (FGFR) 1**: 5'-GCA TGG AGT ATC TGG CCT CC-3' and 5'-GTC CAG GGG CAT GGA CAG-3'; **IL1-β**: 5'-CAG AAG TAC CTG AGC TCG CC-3' and 5'-GGT CGG AGA TTC GTA GCT GG-3';**IL-3**: 5'-GAC TCC AAG CTC CCA TGA CC-3' and 5'-GAT TGG ATG TCG CGT GGG T-3'; **IL-4**: 5'-ACC GAG TTG ACC GTA ACA GAC-3' and 5'-CAG GAA TTC AAG CCC GCC AG-3'; **IL-6**: 5'-TCA GCC CTG AGA AAG GAG ACA T -3' and 5'-TGG CAT TTG TGG TTG GG TCA-3'; **IL8**: 5'-GGG CCA AGA GAA TAT CCG AAC T-3' and 5'-GAG AAA CCA AGG CAC AGT GGA-3'; **IL1 receptor (IL1R) 1**: 5'-GGA GAC GGA GGA CTT GTG TG-3' and 5'-ACT GGC CGG TGA CAT TAC AG-3'; **IL6R**: 5'-AGT GTC GGG AGC AAG TTC AG-3' and 5'-AGT GTC GGG AGC AAG TTC AG-3'; **immunoglobulin-like and EGF-like domains (TIE) 1**: 5'-AGC GCT TCT TCC TGA CTT GC-3' and 5'-GAT GGT GGC TGC ACA TTT GG-3'; **TIE2**: 5'-GGG GAG ATG TGT GAT CGC TT-3' and 5'-TTT GGA TGG AGC ACT GTC CC-3'; **inhibitor of NFκB (IκB)**: 5'-GAA GTG ATC CGC CAG GTG AA-3' and 5'-CTG CTC ACA GGC AAG GTG TA-3'; **intercellular adhesion mlecule (ICAM) 1**: 5'-CTG TGA CCA GCC CAA GTT GT-3' and 5'-GTT CCA CCC GTT CTG GAG TC-3'; **MMP-1**: 5'-TGT GGT GTC TCA CAG CTT CC-3' and 5'-TTG TCC CGA TGA TCT CCC CT-3'; **MMP-2**: 5'-CTT CCG TCT GTC CCA GGA TG-3' and 5'-CAC AGT CCG CCA AAT GAA CC-'3; **matrix metalloprotease (MMP) 9**: 5'-ACC TCG AAC TTT GAC AGC GA-3' and 5'-GCA CAG TAG TGG CCG TAG AA-3'; **N-cadherin**: 5'-AGG CTT CTG GTG AAA TCG CA-3' and 5'- GGA GGG ATG ACC CAG TCT CT-3'; **neuropilin (NRP) 1**: 5'-GCA AAA CCA GCA GAC CTG GA-3' and 5'-TGG TGA TGA GGA TGG GGT CT-3'; **NFκB**: 5'-TTT CAA CCA CAG ATG GCA CT-3' and 5'-AAC CTT TGC TGG TCC CAC AT-3'; **nucleolin**: 5'-GGA AGG CAC AGA ACC GAC TA-3' and 5'-TGT TCT CGC ATC TCG CTC TT-3'; **plasminogen activator inhibitor-1 (PAI-1**): 5'-CTC TCT CTG CCC TCA CCA AC-3' and 5'-AAG GAC TGT TCC TGT GGG GT-3'; **platelet endothelial cell adhesion molecule (PECAM) 1**: 5'-TTT TGG ACC AAG CAG AAG GCT A-3' and 5'-ACG TCT GAG TTC AGA GGC TCT TT-3'; **protease activated receptor (PAR) 1**: 5'-CCT GCT TCA GTC TGT GCG G-3' and 5'-CTG GTC AAA TAT CCG GAG GCA-3'; **P-selectin**: 5'- GCT GGA ATG CTT GGC TTC TG -3' and 5'- TGA

GCG GAT GAA CAC AGT CC -3’; **syndecan-1**: 5′-GCT CAC ACA CCT GTA GCA CT-3′ and 5′-TGC TGT CTC CCG ACC ATA GA-3′; **syndecan-4**: 5′-CCC CAA GAG AAT CTC ACC CG-3′ and 5′-ATT GGT GGG GGC TTT CTT GT-3’; **thrombospondin-1**: 5’-GAG AAT GCT GTC CTC GCT GT-3’ and 5’-TTC TGT ACC CCT CCT CCA CA-3’; **tissue factor**: 5’-CCC AAA CCC GTC AAT CAA GTC-3’ and 5’-CCA AGT ACG TCT GCT TCA CAT-3’; **tissue factor pathway inhibitor (TFPI)**: 5’-CAT TCA AGG CGG ATG ATG GC-3’ and 5’-CAC CAT GAG GGA CCG TGA AA-3’; **tissue-type plasminogen activator (TPA)**: 5’- GGG TGG AAT ATT GCT GGT GC-3’ and 5’-GTA GCA CCA GGG CTT TGA GT-3’; **transforming growth factor (TGF) β**: 5’-GGA AAT TGA GGG CTT TCG CC-3’ and 5’-GAA GTT GGC ATG GTA GCC CT-3’; **thrombomodulin**: 5’-CTA CTG GTC TTG TGG AAT GGG AG-3’ and 5’-AAA GGC CTC TCT GAA ATG GTA GAA A-‘3; **urokinase receptor (uPAR)**: 5’-AGC CTT ACC GAG GTT GTG TG-3’ and 5’-CAG GAT TGG GCC CTC GTT G-3’; **vascular cell adhesion protein 1 (VCAM-1)**: 5’-ACG AAT GAG GGG ACC ACA TC-3’ and 5’-TACAGCCTGCCTTACTGTGG-3’, **VE-cadherin**: 5’-GCA TCG GTT GTT CAA TGC GT-3’ and 5’-TGT GTA CTT GGT CTG GGT GAA G-3’; **vascular endothelial growth factor (VEGF) C**: 5’-GGA CCA AAC AAG GAG CTG GA-3’ and 5’-GTT CGT ACA TGG CCG TCT GT-3’; **VEGF receptor (VEGFR) 3**: 5’-CCA TGA CCC CAA CGA CCT AC-3’ and 5’-CCC TGG TCA CAG CCA CAT TC-3’; **von Willebrand factor (vWF)**: 5’-TGG TGC AGG ATT ACT GCG GC-3’ and 5’-GCT TTG CCC AGC AGC AGA AT-3’; **VEGFA**: 5’-TCA CCA TGC AGA TTA TGC GGA-3’ and 5’-CTC CAG GGC ATT AGA CAG CA-‘3; **VEGF-R1**: 5’-GCA AAG CCA CCA ACC AGA AG-3’ and 5’-ACG TTC AGA TGG TGG CCA AT-3’; **VEGF-R2**: 5’-CGT GTC TTT GTG GTG CAC TG-3’ and 5’-GGT TTC CTG TGA TCG TGG GT-3’.

Gene expression was normalized to β-actin mRNA levels for each sample and the ΔΔct method was used to calculate relative changes of gene expression.[1]

Flow cytometry

The surface expression of TF, thrombomodulin (TM) and VCAM-1 on HUVECs cells was quantified by flow cytometry. Cells were harvested with Accutase (Sigma-Aldrich, Steinheim,

Germany) and incubated with 1 µg of a primary mouse anti-human TF, mouse anti-human TM (American Diagnostica, Stamford, CT) or rabbit anti-human VCAM-1 (Abcam, Cambridge, United Kingdom) or the isotype-specific antibodies in HBRS + 1% bovine serum albumin for 1 hour on ice. The cells were washed and incubated with a phosphatidylethanolamine-conjugated secondary antibody (1:50) for 30min on ice, in the dark. Consecutively, 10 000 cells were analysed using FACS Canto and FACS Diva software (Becton Dickinson, Heidelberg, Germany).

Light transmission aggregometry

Platelet aggregation was evaluated by light transmission aggregometry using a spectrophotometer (PowerWave XS2, BioTek Instruments, USA). Washed platelets were prepared from citrated whole blood as previously reported.[2] Prior to the measurements, 1µl $CaCl_2$ and $MgCl_2$ were added to the platelet suspension (100µl) to a final concentration of 2mM. T24 SN (100µl) with or without IL-1ra or IL-1β diluted in starvation medium was added to the platelet suspension and light transmission was recorded at a wavelength of 600nm for 30min at 37°C.

Transcriptome analysis

To investigate whether UBC affected expression levels of IL-1β at the site of the tumour, we analysed mRNA data from more than 160 bladder cancer patients shared by a publicly available database.[3, 4] Gene transcription levels were correlated with muscle invasiveness, grading and progression.

References

1. Livak KJ, Schmittgen TD. Analysis of relative gene expression data using real-time quantitative PCR and the 2(-Delta Delta C(T)) Method. Methods 2001, 25(4):402-408.
2. Kalagara T, Moutsis T, Yang Y, Pappelbaum KI, Farken A, Cladder-Micus L et al. The endothelial glycocalyx anchors von Willebrand factor fibers to the vascular endothelium. Blood Adv 2018, 2(18):2347-2357.

3. Kim W-J, Kim E-J, Kim S-K, Kim Y-J, Ha Y-S, Jeong P et al. Predictive value of progression-related gene classifier in primary non-muscle invasive bladder cancer. Molecular cancer 2010, 9(1):3.
4. Cerami E, Gao JJ, Dogrusoz U, Gross BE, Sumer SO, Aksoy BA et al. The cBio Cancer Genomics Portal: An Open Platform for Exploring Multidimensional Cancer Genomics Data. Cancer Discov 2012, 2(5):401-404.
