# Supplementary material for: Bladder cancer-derived interleukin-1 converts the vascular endothelium into a pro-inflammatory and pro-coagulatory surface
Source: BMC Cancer. 2020 Dec 2;20:1178. doi: 10.1186/s12885-020-07548-z (PMC7709388; doi:10.1186/s12885-020-07548-z)
Supplement: Supplementary file 2 — Additional file 2: Fig. S1. FACS analysis of HUVEC surface molecules after stimulation with T24 SN. Fig. S2. Platelet binding to T24 cell SN activated endothelial cells. [file 12885_2020_7548_MOESM2_ESM.pdf]

Bladder cancer-derived interleukin-1 converts the vascular endothelium into a pro-inflammatory and pro-coagulatory surface

A. John[1], C. Günes[1], C. Bolenz[1], S. Vidal-y-Sy[2], A. T. Bauer[2], S.W. Schneider[2], C. Gorzelanny[2*]

[1] Department of Urology, University of Ulm, Ulm, Germany

[2] Department of Dermatology, University Medical Center Hamburg-Eppendorf, Hamburg, Germany

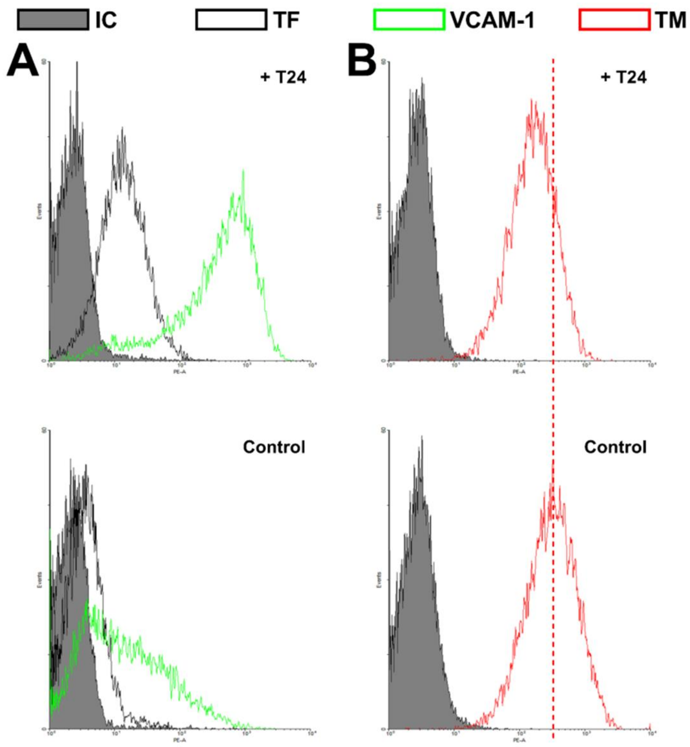

Supplemental Figure 1: FACS analysis of HUVEC surface molecules after stimulation with T24 SN. A Procoagulatory TF and cell-adhesive VCAM-1 were upregulated after 12h incubation with T24 SN. **B** Anticoagulatory thrombomodulin (TM) was reduced. The red dashed line in **B** was added for clarity reasons. **IC:** Isotype control.

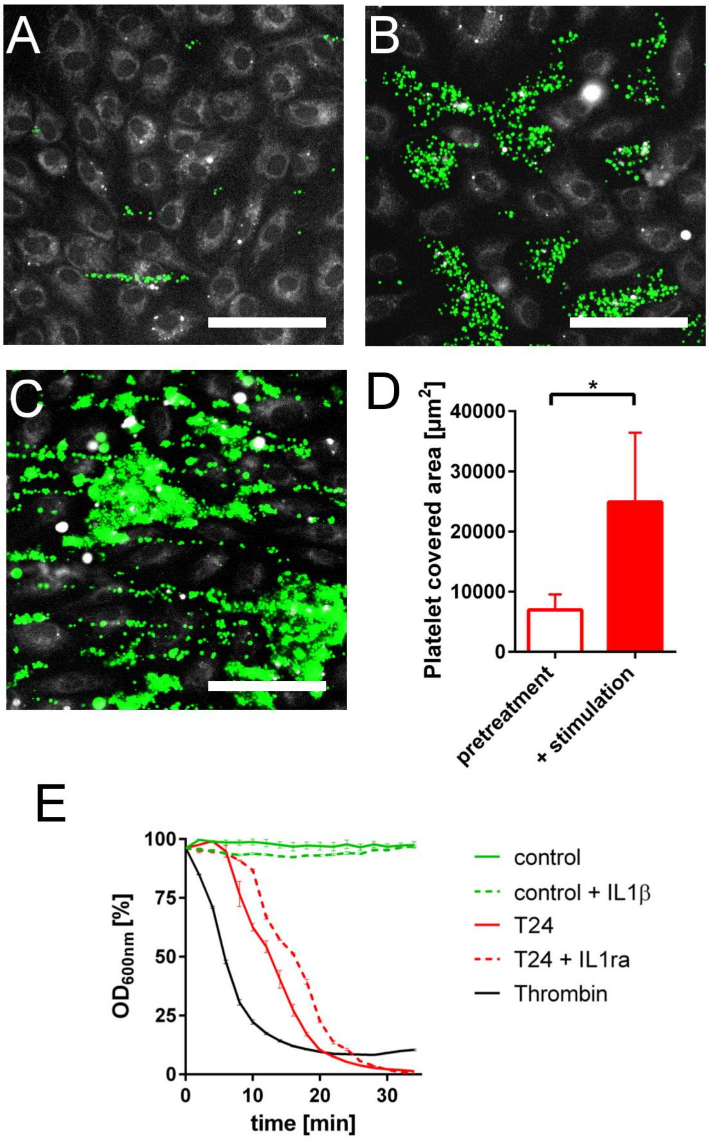

Supplemental Figure 2: Platelet binding to T24 cell SN activated endothelial cells. A-C Representative snapshots of platelets (green) bound to HUVECs (white) taken during microfluidic experiments. HUVECs were perfused with washed and fluorescently labeled platelets at a shear stress of 6 dyn cm^{-2}. Scale bars correspond to 100 µm. **A** HUVECs were pretreated for 6h with control medium. **B** HUVECs were pretreated with T24 SN for 6h. **C** HUVECs were pretreated with T24 SN for 6h and perfusing platelets were stimulated by resuspending them in T24 SN. **D** Quantitative analysis of the platelet binding to T24 SN pretreated HUVECs (pretreatment) and to pretreated HUVECs additionally perfused with platelets resuspended in T24 SN (+ stimulation). **E** Light transmission aggregometry of platelets stimulated with T24 SN (T24), control medium (control), T24 SN supplemented with 30ng/ml IL1ra (T24 + IL1ra), control medium supplemented with 30 ng/ml IL1ϐ (control + IL1 ϐ) or 0.5 U/ml thrombin. Light transmission was measured by a wavelength of 600 nm; data were normalized to the maximum optical density (OD) (100%) and to the least OD (0%).

Supplemental Video 1: Live reflection interference contrast microscopy of HUVECs perfused with whole hirudinated blood. HUVECs were pretreated with T24 cell supernatant for 6 h and subsequently perfused for 15 minutes with whole blood at a shear stress of 6 dyn cm^{-2}. The white arrow indicates the direction of flow. The length of the arrow corresponds to 50µm. Indicated time in mm:ss.
